# Contact Pathway Function During Human Whole Blood Clotting on Procoagulant Surfaces

**DOI:** 10.3389/fmed.2018.00209

**Published:** 2018-07-23

**Authors:** Shu Zhu, Bradley A. Herbig, Xinren Yu, Jason Chen, Scott L. Diamond

**Affiliations:** Department of Chemical and Biomolecular Engineering, Institute for Medicine and Engineering, University of Pennsylvania, Philadelphia, PA, United States

**Keywords:** microfluidics, factor XIa, polyphosphate, platelet, hemodynamics

## Abstract

Microfluidic thrombosis assays allow the control of anticoagulation, hemodynamics, pharmacology, and procoagulant surfaces containing collagen ± tissue factor (TF). With corn trypsin inhibitor (CTI) ranging from low (1–4 μg/mL) to high levels (40–60 μg/mL), the function of Factor XIIa (FXIIa) can be modulated in the presence of low or high surface TF. With high CTI and no collagen/TF in the assay, no thrombin is generated during 15-min microfluidic perfusion. At low CTI (no TF), the generation of FXIa leads to fibrin polymerization at ~300 s after the initiation of flow over collagen, an onset time shortened at zero CTI and prolonged at high CTI. The engagement of FXIa was difficult to observe for clotting on high TF surfaces due to the dominance of the extrinsic pathway. Low TF surfaces allowed observable crosstalk between extrinsic pathway generation of thrombin and thrombin-mediated activation of FXIa, a feedback detected at >5 min and attenuated with polyphosphate inhibitor. From thrombin-antithrombin immunoassay of the effluent of blood flowing over collagen/TF, the majority of thrombin was found captured on intrathrombus fibrin. Additionally, extreme shear rates (>10,000 s^−1^) can generate massive von Willebrand Factor fibers that capture FXIIa and FXIa to drive fibrin generation, an event that facilitates VWF fiber dissolution under fibrinolytic conditions. Finally, we found that occlusive sterile thrombi subjected to pressure drops >70 mm-Hg/mm-clots have interstitial stresses sufficient to drive NETosis. These microfluidic studies highlight the interaction of contact pathway factors with the extrinsic pathway, platelet polyphosphate, VWF fibers, and potentially shear-induced NETs.

## Introduction

**Figure 1:**
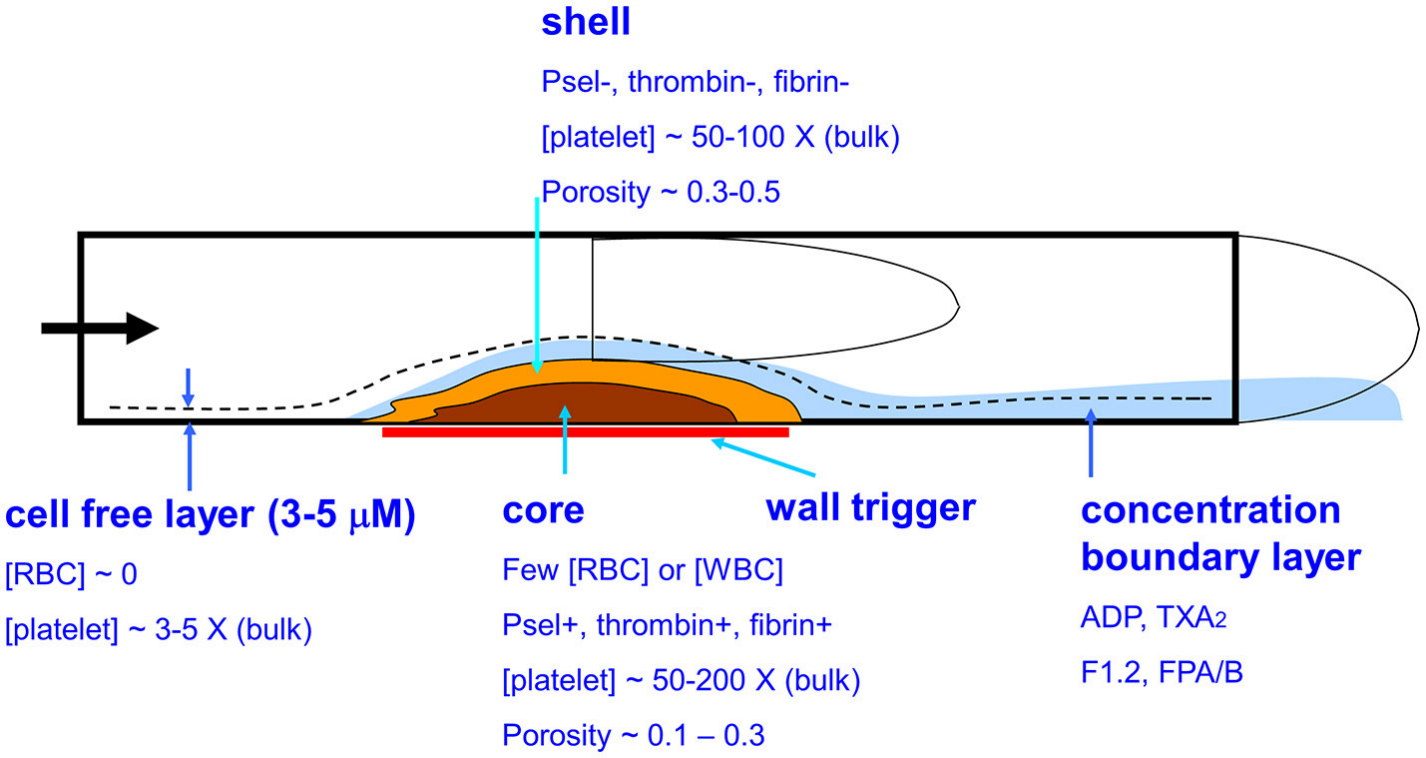
Biophysical and biological aspects of whole blood clotting under flow. Platelets in the cell-free layer flow in close proximity to a prothrombogenic surface resulting in a core-shell architecture where the core is enriched in P-selectin positive platelets, thrombin, and fibrin.

Blood clotting on a thrombogenic surface under flow conditions (Figure 1) is fundamentally distinct from clotting in a test tube (1). In flowing blood, the red blood cells (RBCs) move toward the center of the vessel while platelets accumulate in the plasma layer near the wall (2). Reduction of the hematocrit dramatically reduces the ability of platelets to interact with a thrombogenic surface causing an associated defect in platelet deposition, thrombin generation, and fibrin polymerization (3). Platelets accumulate to high concentration on procoagulant surfaces under venous or arterial flow with relatively few RBCs or neutrophils in the growing thrombus. Over time, the innermost core of platelets become highly activated and P-selectin-positive (4) at the site of thrombin generation and fibrin deposition (5, 6). Surrounding this core is a shell of less activated platelets (P-selectin negative) where ADP and thromboxane play and important role in the growth of the shell (7). A concentration boundary layer of molecules can be found at high concentration on the outer surface of the clot (8) and these constituents move downstream and eventually become mixed into the blood. Small molecules such as ADP, thromboxane, free Zn ions, fibrinopeptides A and B, and Fragment 1.2 (F1.2) are expected to be released into the boundary layer of the clot. Interestingly, little thrombin is released by a clot due to its sequestration by fibrin (9). In terms of thrombosis under flow, platelets and their releasates can accumulate to very high concentrations relative to levels found in platelet-rich plasma (PRP). Additionally, under flow conditions, fresh zymogen factors like prothrombin and fibrinogen can continually enter the clot from the flowing blood to sustain intrathrombus coagulation reactions, a situation very different from clotting in a tube (10).

The study of the contact pathway, specifically FXIIa and FXIa, under *ex vivo* conditions is complicated by several constraints: (i) drawn blood is exposed to metal (syringes), various plastics, and even glass, (ii) platelets and FXII can activate on foreign surfaces, and (iii) drawn blood may harbor tissue factor from the phlebotomy. For microfluidic studies, the priming of PDMS microfluidic channels and glass surfaces with albumin-containing buffers (typically 0.5 to 1% by wt/vol.) will dramatically reduce nonspecific interactions such that few platelets and no fibrin are found in the reservoir and microchannels leading to the collagen feature that triggers and localizes clotting. Additionally, the deployment of corn trypsin inhibitor (CTI) provides relatively specific inhibition of βFXIIa. CTI has a K_i_ of 2 nM against βFXIIa (11) and an IC50 of 110 nM against αFXIIa (12). The αFXIIa form drives surface-dependent activation of FXI, while βFXIIa drives solution phase pre-kallikrein activation. The combined use of albumin buffer priming and 40 μg/mL CTI inhibits thrombin generation to near zero levels for 15 min whole blood perfusions when the microfluidic devices lack a procoagulant surfaces such as fibrillar type 1 collagen (10).

In this paper, we investigate the relative clotting strengths of the contact pathway, the extrinsic pathway, and their crosstalk. We show how CTI can be used to “dial in” the extent of coagulation through the contact pathway when using human blood *ex vivo*. We present data that platelet-derived polyphosphate can promote the thrombin-mediated feedback activation of FXI under flow conditions, a crosstalk that requires a low level of TF to prime the interaction. We further show that the contact pathway tends to exert its influence at later stages of the clotting event, typically after 5 min of clotting. Beyond physiological flows, microfluidic devices allow the study of pathological high shear flows that would exist only in a severe stenosis or in a biomechanical pumping device. Interesting cross-reactions can be observed between the contact pathway, VWF, and tissue plasminogen activator (tPA). Finally, we demonstrate hemodynamic conditions where a pressure-driven Darcy flow through sterile thrombotic occlusions can drive shear-induced neutrophil extracellular traps (NETs) that may have the potential to participate in clotting reactions.

## Materials and methods

### Reagents and blood collection

The following reagents were obtained and stored following manufacturers' instructions: corn typsin inhibitor (CTI, Haematologic Technologies, Essex Junction, VT); type 1 fibrillar collagen (Chronolog Corp, Havertown, PA); lipidated tissue factor (TF, Dade Innovin, Siemens, Malvern, PA); anti-human CD61 antibody (BD Biosciences, San Jose, CA); Alexa Fluor-647 conjugated human fibrinogen (Life Technologies, Grand Island, NY); Sytox green (Life Technologies, Grand Island, NY); thrombin-antithrombin (TAT) ELISA (Abcam, Cambridge, MA). The antibodies O1A6 and 14E11 were kindly provided by Dr. Andras Gruber (Oregon Health and Sciences University). Polyphosphate binding domain (PPXbd), the recombinant polyP-binding domain of *E. coli* exopolyphoshatase, was kindly provided by the James Morrissey Laboratory (Univ. Mich.) Blood was drawn into CTI (low level, 1–4 μg/mL, or high level, 40 μg/mL) from adult male and female donors who provided informed consent under IRB approval (Univ. Penn.) who self-reported free of alcohol or medication use.

### Microfluidics

An 8-channel PDMS microfluidic device was prepared as previously described (13, 14). The microfluidic patterning device (13, 14) is a single channel PDMS device to create a 250-μm wide strip of fibrillar collagen on glass, with or without lipidated TF (low, 0.1 molecule TF/μm^2^; high ~1 molecule-TF/μm^2^) (14) or kaolin. The micropatterning device is easily removed from the glass slide without disturbing the patterned collagen. Nominal concentration of surface TF were determined using measured surface coverage, average liposomal radius (118 nm) with 20 molecules/liposome, and assumed 50% incorporation of TF with the extracellular domain facing the bulk fluid. Experimentally, the concentration of 2 molecules-TF/μm^2^ is on the high end of the dose-response curve with higher concentrations not generating fibrin sooner or more abundantly. Kaolin surface concentration was measured as (15) where fluorescently-labeled kaolin particles were visualized with fluorescent microscopy and found to be fully resistant to any flow washout at 1,000 s^−1^. The 8-channel device was positioned on the glass such that each channel (60 μm high by 250 μm wide) ran perpendicularly across the patterned procoagulant surface. CTI-treated whole blood was perfused at venous or arterial shear rates while platelet and fibrin deposition were measured in real time by fluorescence microscopy.

## Results

### Clotting on kaolin/collagen surface

**Figure 2:**
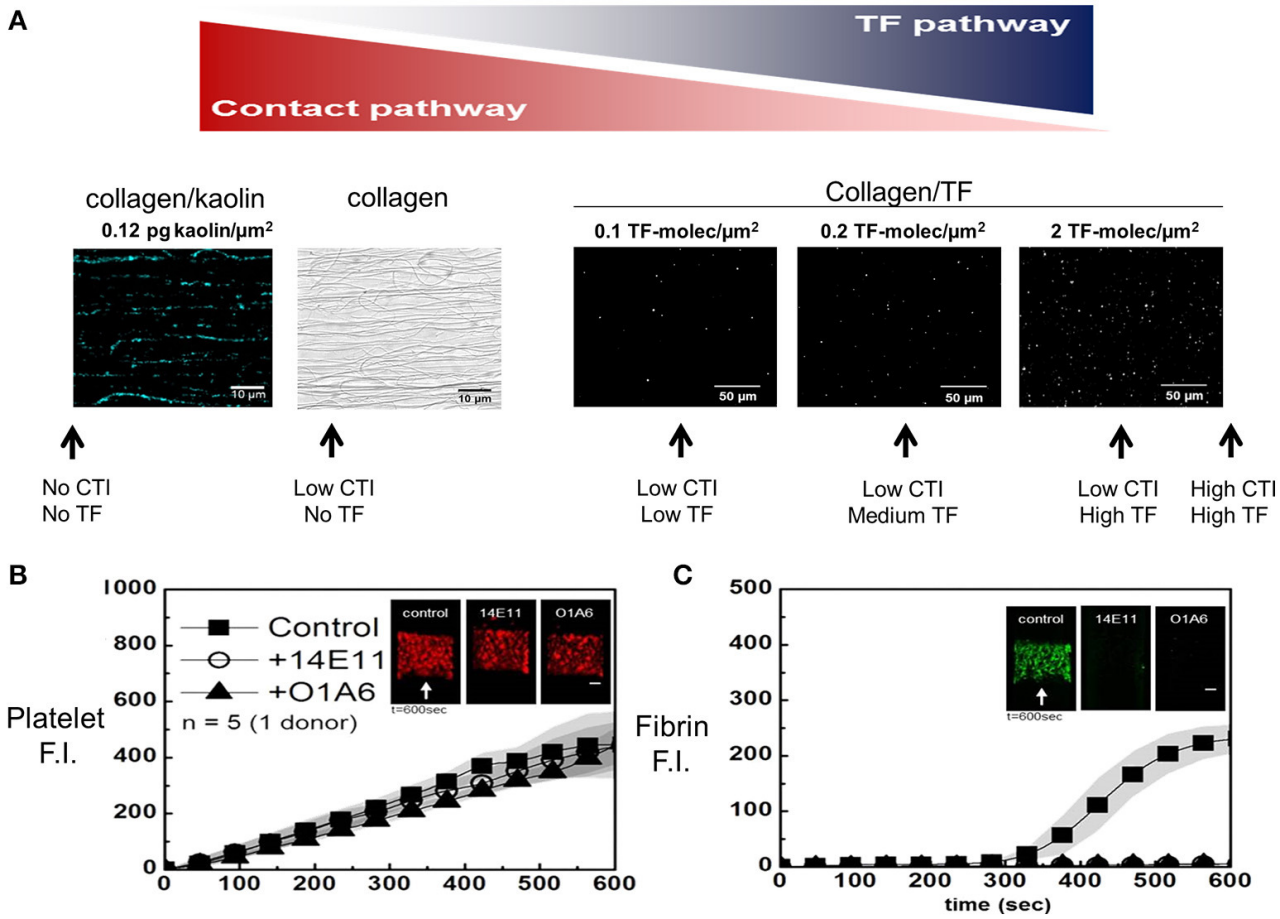
Micropatterned surface **(A)** of fibrillar collagen can be patterned along with a contact activator (kaolin in blue, left) or an extrinsic activator (lipidated TF in white, right). The fluorescently labeled kaolin particles were visualized (**A**, left) (15), while FITC-annexin V allowed staining of sorbed lipidated TF at increasing concentrations (**A**, right). The amount of TF (or kaolin) and the amount of CTI are shown schematically (**A**, arrows) to illustrate different experimental conditions to modulate the amount of contact pathway and extrinsic pathway triggering of clotting. In the absence of surface TF, without affecting platelet deposition **(B)**, all the fibrin produced is the result of FXIa activity **(C)**, which is blocked by 20 μg/mL of O1A6 or 14E11 at 100 s^−1^ (14).

**Figure 3:**
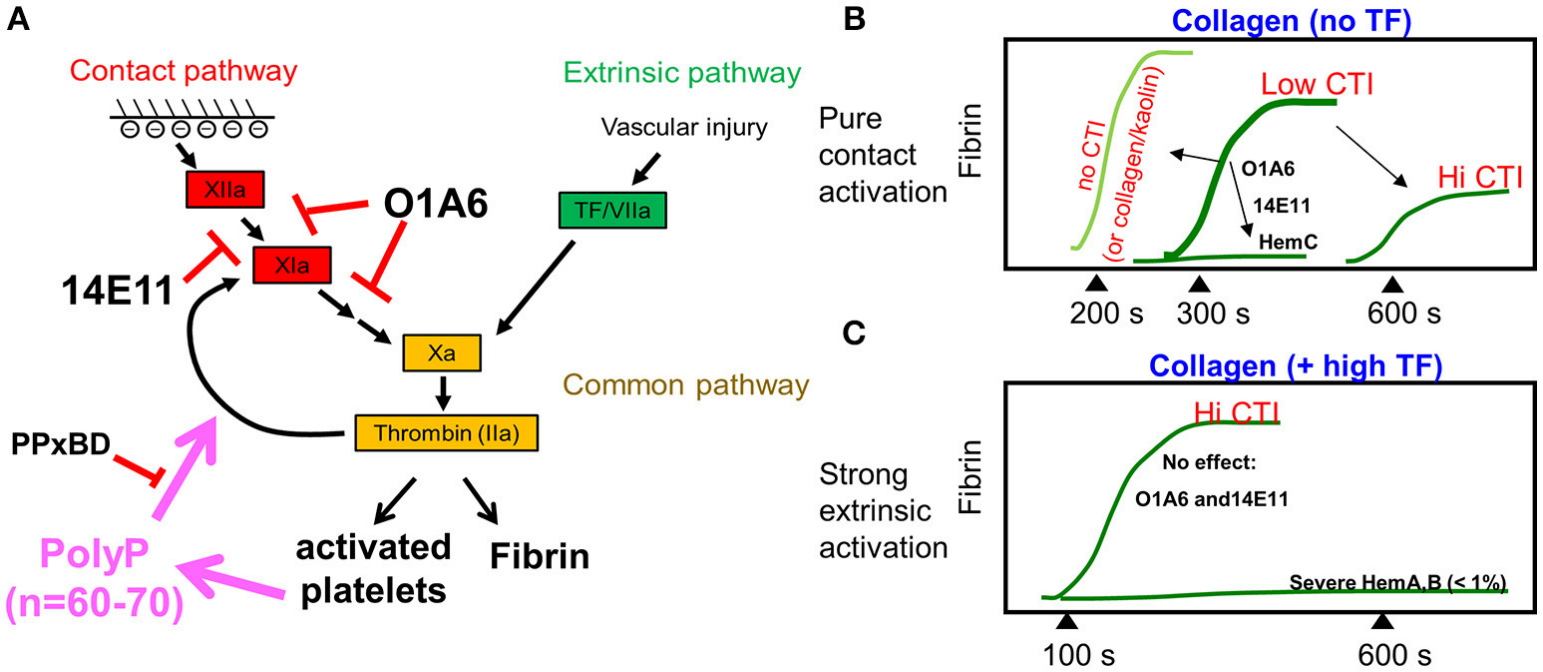
Schematic diagram summarizing the thrombin generation and fibrin generation through the contact pathway or the extrinsic pathway **(A)**. FXIa antibodies or increasing amounts of CTI delay fibrin generation for whole blood clotting under flow, while FXI-deficiency (hemophilia C, hemC) prevents fibrin formation (**B**, top). Fibrin generation during whole blood flow over collagen/high TF is not affected by FXIa inhibition, but requires intrinsic tenase (FIXa/FVIIIa) **(C)**.

Kaolin and silica are common laboratory reagents for activating the contact pathway. It is reasonable to hypothesize that these powders are also physiological activators of FXII in terrestrial mammals requiring wound hemostasis/infection control in the presence of dirt. Interestingly, fish lack FXII and certain ocean mammals have lost FXII expression (16). Placement of kaolin or TF into a collagen surface, along with appropriate choice of the CTI concentration in the perfused blood, allows a titration from full contact activation to full extrinsic activation of blood clotting (Figure 2A). This approach also allows for intermediate regimes where both the contact and extrinsic pathways can contribute to initiation and propagation of clotting. For patterned kaolin (0 to 0.3 pg/μm^2^)/type 1 collagen fibril surface and venous low-CTI whole blood perfusion (wall shear rate, 100 s^−1^), the initiation of fibrin formation occurred by ~100 s faster when compared to collagen alone (~ 350 s). Also, a TF/collagen surface produces fibrin earlier and more abundantly than a kaolin/collagen surface. Both triggering surfaces had a similar sensitivity for reduced fibrin deposition at arterial wall shear rates of 1,000 s^−1^, compared to fibrin formed at a venous shear rate on either surface [see Figures 3C,D in 15].

Perfusion of low CTI-treated whole blood over pure collagen (no kaolin and no TF) results in a continuous accumulation of platelets (Figure 2B) and an onset of fibrin deposition starting at about 300 s (Figure 2C). In some experiments, anti-FXI antibodies (14E11 and O1A6) were used to block FXIIa-dependent FXI activation or FXIa-dependent factor IX (FIX) activation, respectively. In this assay, the accumulation of platelets was largely driven by the strength of the fibrillar collagen surface to drive activation (via GPVI signaling) and secondary deposition (ADP/thromboxane/α_IIb_β_3_-dependent), and was essentially independent of FXIa-inhibiting antibodies. In contrast, fibrin deposition was fully blocked by 14E11 or O1A6 antibodies targeting FXIa production and function. Thus, thrombin and fibrin generation for low CTI-whole blood flow over pure collagen is entirely dependent on the contact pathway, with no evidence for kinetically significant bloodborne TF in healthy donor blood.

Several clotting studies (14, 15, 17) that explore the presence and absence of surface TF under different clotting conditions and blood genotypes are summarized in Figure 3. In the absence of added surface TF, fibrin first appears at about 300–350 s and is fully blocked by anti-FXI antibodies (Figure 2B) or by severe human Factor-XI deficiency (17) (Figure 3B). The absence of CTI (raw blood perfusion) results in fibrin onset at about 200 s, similar to observations with kaolin-presenting surfaces perfused with low CTI-treated whole blood (15). In contrast, perfusion of high CTI-treated whole blood over TF-presenting collagen allows rapid engagement of the extrinsic pathway with robust fibrin production within about 100–150 s (Figure 3C). With sufficient TF on the surface (typically >1 molecule-TF/μm^2^), the engagement of the contact pathway is difficult to observe since the extrinsic pathway can produce ample thrombin, a potential limitation for FXIIa/FXIa inhibition therapies against TF-laden plaque rupture events. Under this condition of high TF on a surface, the extrinsic tenase activity of TF/FVIIa is not sufficient for the robust FXa and thrombin generation needed for fibrin generation. Severe hemophilia A or B blood produces little fibrin on TF-presenting surfaces (18), demonstrating the importance of the intrinsic tenase (FIXa/FVIIIa) for robust generation of FXa to make enough thrombin to polymerize fibrin. In general, the extrinsic tenase produces signaling levels of FXa (and subsequently sub-nM signaling levels thrombin) to activate platelet PARs, while the intrinsic tenase is needed to generate clotting levels of FXa to make enough thrombin (>10 nM) to make fibrin. Clotting on TF is always more rapid and robust than clotting that depends only on the contact pathway.

### Role of platelet polyphosphate during clotting under flow

**Figure 4:**
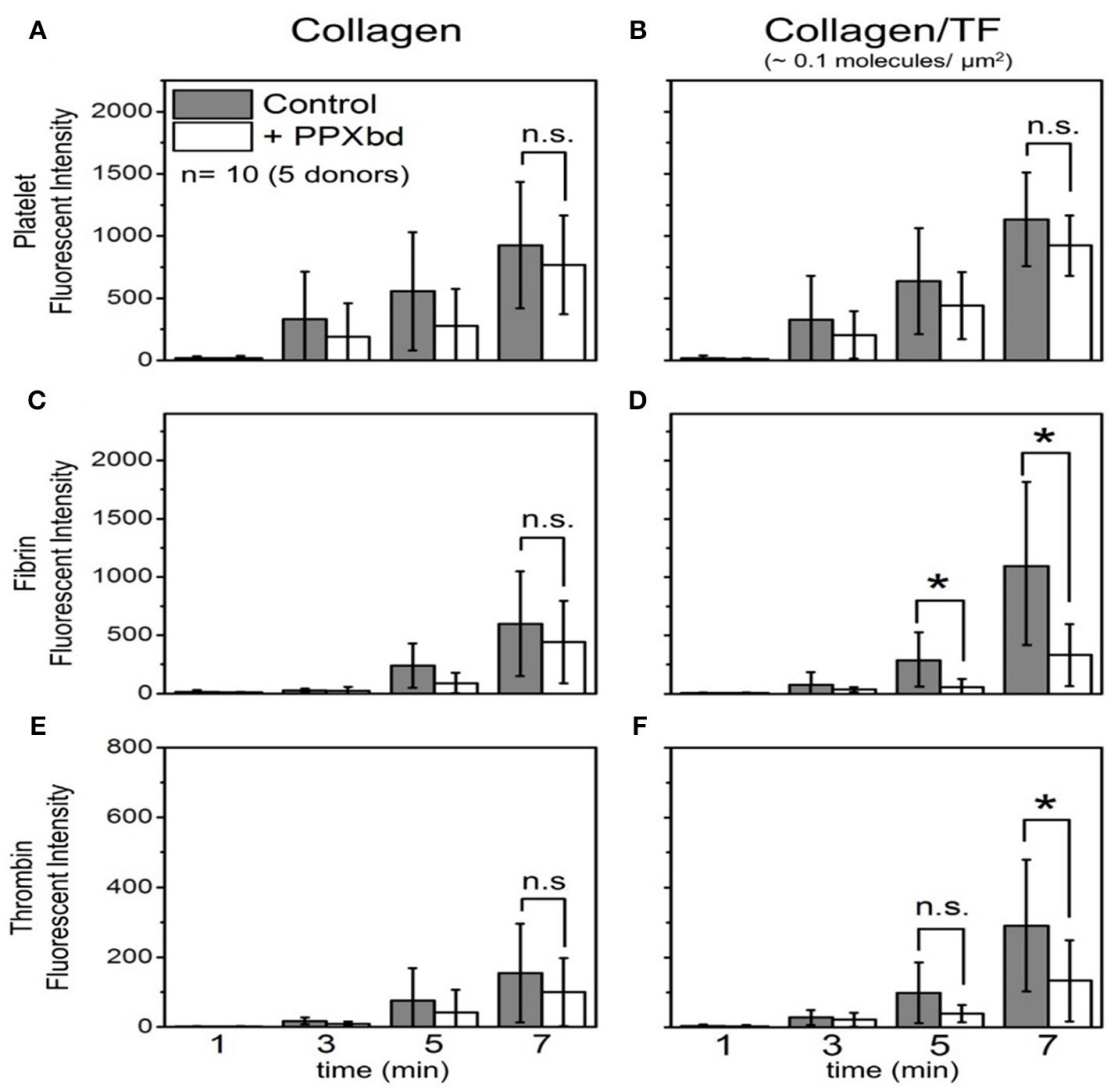
Platelet deposition, thrombin generation and fibrin polymerization on collagen **(A,C,E)** or collagen/TF **(B,D,F)** in the absence or presence of a polyphosphate inhibitor (PPxBD) (14). *P < 0.05.

Since platelets accumulate under flow to such high concentration on a collagen surface (>50-200X PRP concentrations), any clot-captured releasate from those platelets will reach local concentrations in the clot that greatly exceed the levels in closed system tube experiments. The addition of a polyphosphate inhibitor to low-CTI whole blood perfused over a low TF surface had little effect on platelet deposition but reduced thrombin generation [indicated by a platelet-targeted biosensor 5] and fibrin deposition by about 50% (Figure 4). PPXbd reduced fibrin generation at low [TF]_wall_ (0.1 molecules per μm^2^) but not at zero or high [TF]_wall_ (>1 molecules per μm^2^), suggesting a role for polyP distinct from FXIIa activation and requiring low extrinsic pathway participation. Interestingly, at 0.2 molecule-TF/μm^2^ and 100 s^−1^, the antibody 14E11 did not significantly reduce fibrin formation while O1A6 did [see Figure 2E in 9]. This effect of PPXbd was not seen with high TF presenting surfaces since the extrinsic pathway dominates the thrombin production.

### Dynamics of thrombin production under flow

**Figure 5:**
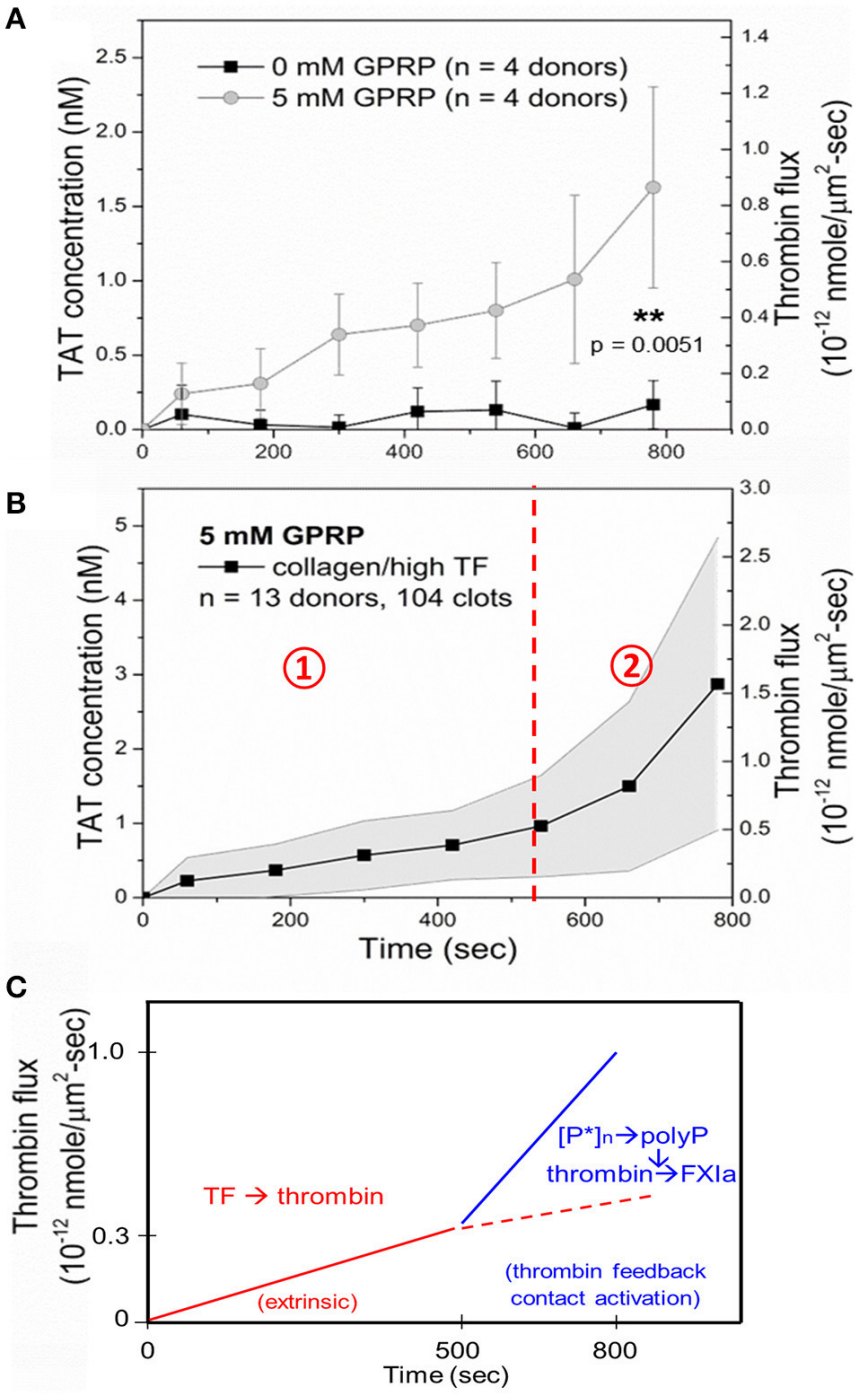
Measurement of thrombin-antithrombin (TAT) in the effluent allows calculation of thrombin flux per unit area per unit time for a collagen/TF surface. TAT is largely undetectable unless fibrin polymerization is inhibited with GPRP **(A)**. The average thrombin flux for whole blood clotting in the presence of GPRP **(B)**. Role of polyphosphate and FXIa in late stage thrombin generation at times > 500 s **(C)** (9).

Few measurements have been made of the amount of thrombin generated during whole blood clotting under flow conditions. Using TAT ELISA, the effluent of blood flowing over a collagen/TF surface, we were able to calculate the dynamics of thrombin generation. Little thrombin actually leaves the clot when fibrin is allowed to form. This is fully consistent with the observed role of gamma-prime fibrinogen exerting antithrombin I activity (6). Using Gly-Pro-Arg-Pro (GPRP) to block fibrin polymerization, thrombin does elute from the platelet deposit, allowing a calculation of the flux of thrombin per unit area from the thrombogenic surface (Figure 5A). In the absence of fibrin polymerization, the thrombin flux increased linearly with time to a level of 0.5 × 10^−12^ nmol/μm^2^-s by 500 s, and then increases about 3-fold over the next 300 s due to generation of Factor XIa, as indicated by a blockade of the late stage enhancement with FXIa-antibodies (Figure 5B) (6). Similar results have been obtained using F1.2 generation in the presence of fibrin polymerization (10). Since the flux of thrombin has been measured and most of it is captured within fibrin in the core of the clot, it is possible to estimate the intrathrombus concentration of thrombin in the core to be a striking 10 μM concentration although the majority of this thrombin (~99%) is bound to fibrin based upon equilibrium binding with the strong and weak site.

Overall, the clotting process on a TF-rich surface is dominated in the first 500 s by the extrinsic pathway and platelet polyphosphate and FXIa-dependent pathways, leading to enhanced fibrin production at later times of 500 to 800 s of clotting (Figure 5C). Using F1.2 generation as a metric of thrombin generation in the presence of fibrin polymerization, FXIa-antibodies had similar late stage inhibition of fibrin polymerization at > 500 s of clotting as since with the TAT assay using GPRP (9, 10, 14).

### VWF fiber formation at pathological shear flows

**Figure 6:**
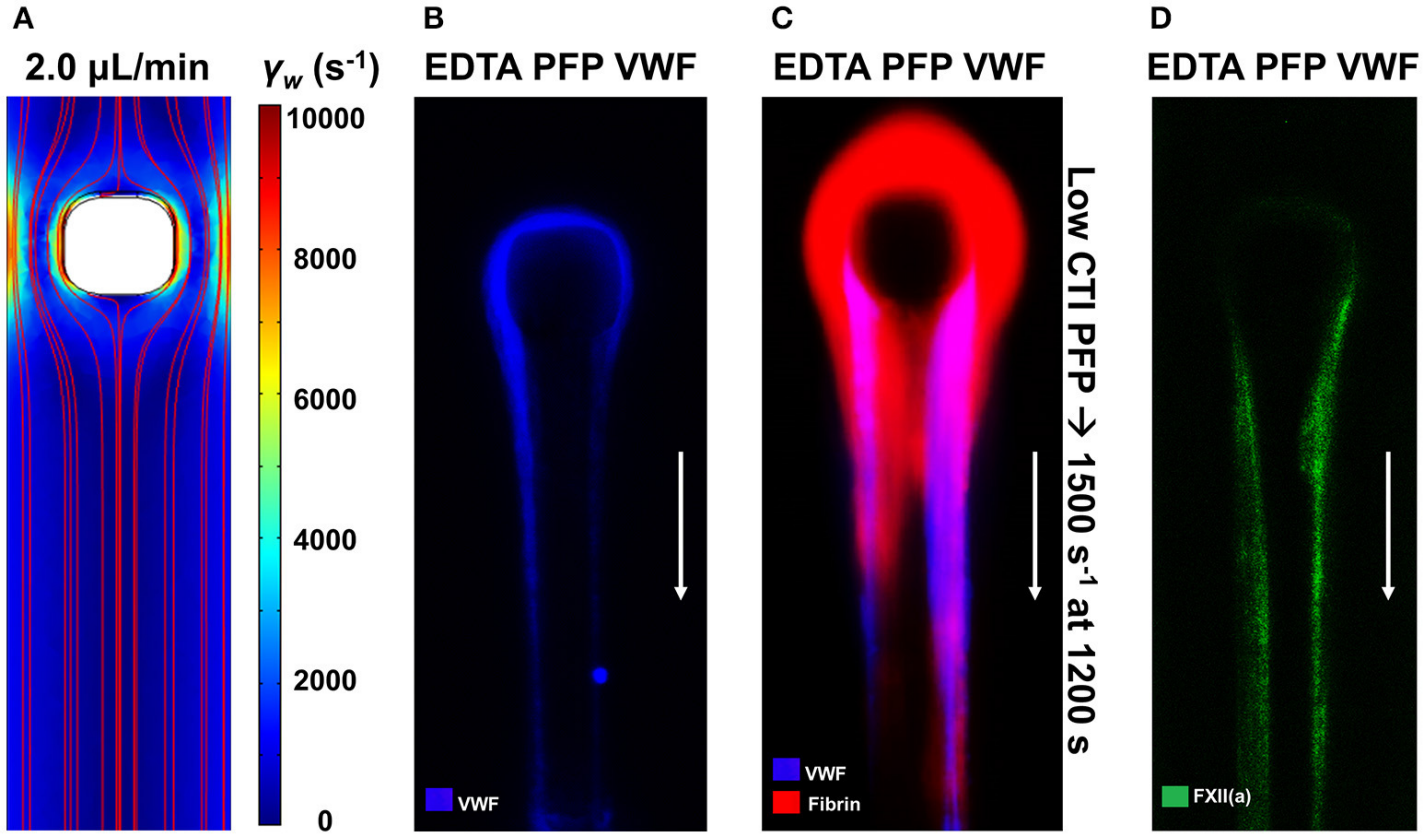
VWF fibers promote contact activation via Factor XII capture. The impingement-post microfluidic device contains a stenotic channel with a micropost in flow to capture aggregated VWF fibers, which are held in place by converging flows downstream of the micropost **(A)**. VWF fibers are captured on the naked micropost by perfusing EDTA-inhibited platelet-free plasma (PFP) at an upstream wall shear rate of 10,000 s^−1^ **(B)**. After formation of VWF fibers, perfusion of coagulable low-CTI-inhibited PFP at 1,500 s^−1^ results in fibrin formation on the VWF fibers **(C)**. Using a fluorescent antibody against FXII(a), FXII(a) was found to colocalize with VWF fibers **(D)** (20).

The mechanobiology of VWF and platelet GPIba has revealed numerous interesting aspects of clotting under flow conditions. At pathological conditions found in coronary stenosis, VWF can undergo a coil-stretch transition. VWF from plasma forms dense and long VWF fiber assemblies when plasma is perfused over collagen at high wall shear rates (19). In an assay that lacks collagen, plasma VWF can form fibers using an impingement-post microfluidic device (20, 21). These VWF fibers rapidly dissolved in trypsin, plasmin or 2% SDS, but were resistant to 50 nM ADAMTS13 or 100 nM tPA in calcium-containing apixaban/PPACK-treated plasma. Additionally, these fibers can then be perfused with low CTI plasma that allows FXIIa production. Fibrin formation on VWF fibers was blocked by anti-FXI antibody 14E11 [see Figure S6 of 20] demonstrating that VWF-localized FXIIa was generating FXIa, ultimately to lead to fibrin deposition on the VWF fiber (Figure 6). The FXIIa is activated elsewhere in the system in low CTI-plasma and then captured onto the VWF fibers [see Figure 4D of (20)].

### Shear-induced netosis in sterile occlusive thrombi

**Figure 7:**
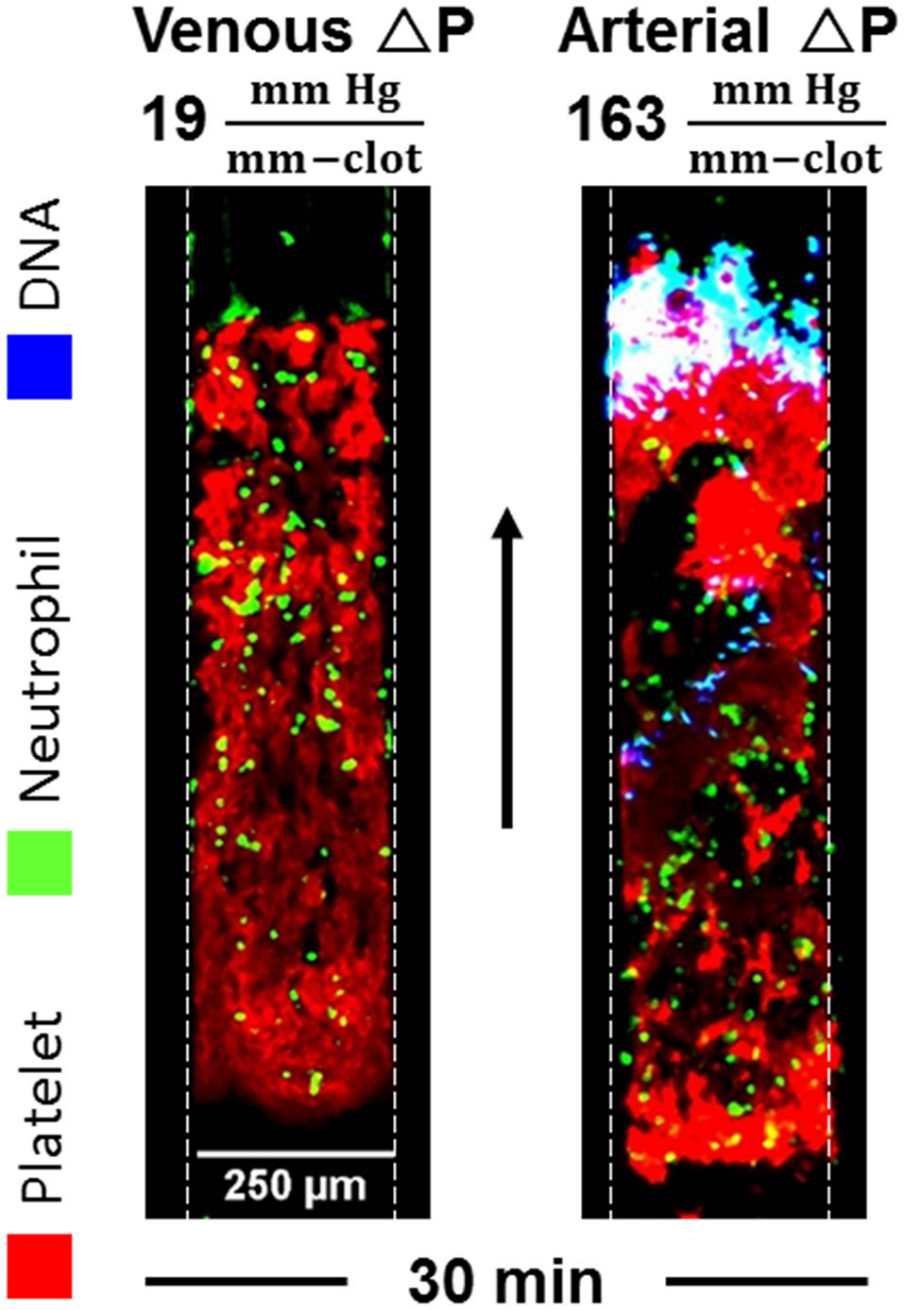
NETs are detected within sterile thrombotic occlusions formed on collagen or collagen/TF. NETs only occur when the transthrombus pressure-drop exceeds 70 mm-Hg/mm-clot, a condition associated with various angiopathies (24).

Neutrophil extracellular traps (NETs) are reported to contain components that activate the contact pathway, although these pathways are not fully resolved (22). Caution is required in concluding that nucleic acid is a contact activator since silica particles co-elute with DNA obtained from commonly used commercial columns (23). We have used microfluidic assay to understand the generation of NETs during sterile thrombosis (24). Using Sytox Green, we imaged NETs during perfusion of CTI-inhibited or PPACK-inhibited human whole blood over fibrillar collagen (±TF) at venous and arterial flow conditions. Platelets rapidly accumulated and occluded microchannels with a late stage infiltration of neutrophils. However, NETosis was detected only in the arterial condition where significant Darcy permeation was driven by a pressure drop in excess of 70 mm-Hg/mm-clot (Figure 7). The level of shear-induced NETs (SINs) at 30 min was >150-fold higher in the arterial condition in the absence of thrombin and >80-fold greater in the presence of thrombin than the level in the venous condition. The observed NETs contained citrullinated histone H3 and myeloperoxidase, and were DNase-sensitive. For measured pressure gradients exceeding 70 mm-Hg/mm-clot to drive interstitial flow, the calculated fluid shear stress on neutrophils exceeded the known lytic value of 150 dyne cm^−2^. Shear-induced NETs may be a biomarker of pathological hemodynamics during arteriole angiopathy. The role of shear-induced NETs in driving contact activation remains to be investigated.

## Discussion

We have explored several aspects of the contact pathway function under flow conditions created in microfluidic devices. The use of albumin-priming of surfaces and high CTI completely blocks thrombin production for over 15 min in devices that do not present a procoagulant collagen surface. CTI at >20 μg/mL is reported to inhibit FXIa (*K*_d_ = 8 μM = 100 μg/mL) (25). However, others found no FXIa-inhibition at up to 300 μg/mL CTI when adding FXIa to FXIIa-deficient plasma (26). Clearly at 4 μg/mL CTI, ample FXIa activity is present to generate fibrin (Figure 2C), an activity strongly inhibited by anti-FXIa (14E11 or O1A6). The Figure 2C result supports the interpretation of Figures 4D,**F** that FXIa is active at the 4 μg/mL CTI condition and that PPXbd interfered with FXIa generation. Separately, the 40 μg/mL CTI condition of Figure 5 allowed the detection of TAT generation under a condition dominated by TF on the surface, however the role of late stage generation of FXIa (at >500 s, Figure 5C schematic) was still detectable when O1A6 was deployed [Figure 3B of (9), and Figure 4B of 10]. This experimental control provides the foundation to reliable study contact pathway *ex vivo* using freshly drawn human blood.

The combined use of high CTI and high TF-laden surfaces allows the study of the extrinsic pathway with little observable contribution of the contact pathway. The use of low CTI with collagen and no TF or low levels of TF on collagen represents a condition where the intrinsic pathway can participate in the clotting episode, particularly at longer times > 500 s where platelet polyphosphate enhancement of thrombin-mediated FXIa generation becomes a kinetically significant pathway. Inhibitors of platelet polyphosphate and FXIa reduce thrombin and fibrin generation at >500 s of clotting, suggesting their utility as antithrombotic agents. The intrathrombus activator(s) of FXII to FXIIa remain to be prioritized with respect to thrombogenic risk in human clotting syndromes, particularly with respect to plaque rupture.

In the context of arterial thrombosis with coronary syndromes, pathologically high shear flows cause VWF to spontaneously form dense and massive VWF fibers. These fibers are substrates for capture of FXIIa and FXIa and the subsequent generation of fibrin. Interesting VWF fibers are not cofactor for tPA-mediated plasminogen activation. However, the fibrin polymerized on VWF allows for the generation of plasmin by tPA, leading to the degradation of co-localized fibrous VWF.

## Author contributions

SZ, BH, XY, and JC conducted all experiments and SD designed the study. All authors contributed to the writing of the manuscript.

### Conflict of interest statement

The authors declare that the research was conducted in the absence of any commercial or financial relationships that could be construed as a potential conflict of interest.
